# A sequential methodology for integral evaluation of motor and non-motor behaviors in parkinsonian rodents

**DOI:** 10.1016/j.mex.2020.100821

**Published:** 2020-02-20

**Authors:** Luis O. Soto-Rojas, Cecilia Bañuelos, Linda Garces-Ramirez, Claudia Luna-Herrera, Yazmin M. Flores-Martínez, Guadalupe Soto-Rodríguez, Bismark Gatica-García, Francisco E. López-Salas, José Ayala-Dávila, María E. Gutiérrez-Castillo, América Padilla-Viveros, Fidel de la Cruz-López, Irma A. Martínez-Davila, Daniel Martinez-Fong

**Affiliations:** a Departamento de Fisiología, Escuela Nacional de Ciencias Biológicas, Instituto Politécnico Nacional, Av. Wilfrido Massieu s/n, Unidad Profesional “Adolfo López Mateos”, Ciudad de México 07738, México; b Facultad de Estudios Superiores Iztacala, Universidad Nacional Autónoma de México, Av. de los Barrios No. 1, Los Reyes Iztacala, Tlalnepantla, Edo. de México 54090, México; c Coordinación General de Programas Multidisciplinarios. Programa Transdisciplinario en Desarrollo Científico y Tecnológico para la Sociedad, Av. Instituto Politécnico Nacional No. 2508, Centro de Investigación y de Estudios Avanzados, Ciudad de México 07360, México; d Departamento de Fisiología, Biofísica y Neurociencias, Centro de Investigación y de Estudios Avanzados, Av. Instituto Politécnico Nacional No. 2508, Ciudad de México 07360, México; e Facultad de Medicina, Benemérita Universidad Autónoma de Puebla, 13 Sur 2702, Puebla 72420, México; f Programa de Doctorado en Nanociencias y Nanotecnología, Av. Instituto Politécnico Nacional No. 2508, Centro de Investigación y de Estudios Avanzados, Ciudad de México 07360, México; g Departamento de Biociencias e Ingeniería, Centro Interdisciplinario de Investigaciones y Estudios sobre Medio Ambiente y Desarrollo, Instituto Politécnico Nacional, 30 de junio de 1520s/n, Ciudad de México 07340, México

**Keywords:** Depressive like-behavior, Locomotor asymmetry, Bradykinesia, Sensorimotor alteration, Uncoordinated gait, Parkinsonism, BSSG, 6-OHDA

## Abstract

An animal model, suitable for resembling Parkinson's disease (PD) progress, should show both, motor and non-motor alterations. However, these features have been scarcely evaluated or developed in parkinsonian models induced by neurotoxins. This protocol provides modifications to original methods, allowing six different motor and non-motor behavior tests, which adequately and timely emulate the main parkinsonian sensorimotor alterations in the rat or mouse: (1) bilateral sensorimotor alterations, examined by the vibrissae test; (2) balance and motor coordination, evaluated by the uncoordinated gait test; (3) locomotor asymmetry, analyzed by the cylinder test; (4) bradykinesia, as a locomotor alteration evidenced by the open field test; (5) depressive-like behavior, judged by the forced swimming test; and (6) hyposmia, assessed by the olfactory asymmetry test. Some advantages of using these behavioral tests over others include:

• No sophisticated materials or equipment are required for their application and evaluation.
• They are used in rodent models for parkinsonian research, but they can also be helpful for studying other movement disorders.
• These tests can accurately discriminate the affected side from the healthy one, after unilateral injury of one hemisphere, resulting in sensorimotor, olfactory or locomotor asymmetry.

## Graphical abstract

**Figure:**
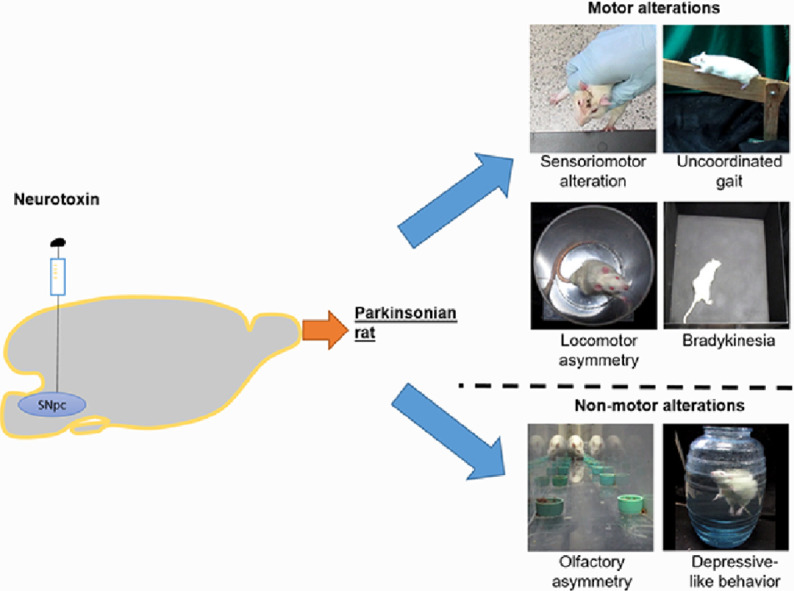

**Table:** Specification Table

Subject Area:	Neuroscience
More specific subject area:	Behavioral neuroscience
Method name:	A sequential methodology for integral evaluation of motor and non-motor behaviors in parkinsonian rodents
Name and reference of original method:	1. Forced swim test (Slattery et al., J Pharmacol Exp Ther 2005:312:1:290–296; Slattery et al., Nat Protoc 2012:7:6:1009–1014). 2. Open field test (Balkaya et al., J Cereb Blood Flow Metab. 2013: 33:3: 330–338; Hernandez et al., J Biomed Sci. 2015:22:59). 3. Tactile Forelimb Placing Tests (Barth, et al., Cerebral Cortex, 1994:4:3:271–278; Woodle, et al., Experimental Neurology 2008:211:2:511–517). 4. Corridor test (Dowd et al., Brain Research Bulletin 2005:68:1–2:24–30; Boix et al., Behav Brain Res 2015:284:196–206). 5. Beam walking (Carter, et al., Journal of Neuroscience 1999:19:8:3248–3257; Luong, et al., J Vis Exp. 2011:10:49.). 6. Limb-use asymmetry (“cylinder”) test (Balkaya et al., J Cereb Blood Flow Metab. 2013: 33:3: 330–338; Woodle, et al., Experimental Neurology 2008:211:2:511–517; Reyes, et al., Plos One 2017:12:11).
Resource availability:	Data are available in the article

## Method details

The methodology presented here allows performing and evaluating motor and non-motor behavioral alterations, resulting in an integral analysis of the PD model. Six different behavioral tests are described step by step and proposed to execute them sequentially, either in a single time or in a temporal course. To exemplify and validate the behavioral tests and show sensorimotor alterations, two different neurotoxins were used to generate independent parkinsonian rat models. We administered β sitosterol d- glucoside (BSSG) into the substantia nigra and 6-hidroxydopamine (6-OHDA) into the striatum. BSSG is a neurotoxin found in the plant *Cycas micronesica* [1], which has been recently used to develop a PD model with construct, face, and predictive validity [2,3]. Meanwhile, 6-OHDA is the most widely used neurotoxin for modeling PD in experimental animals [4,5].

It should be emphasized that the behavioral tests validated here by using BSSG and 6-OHDA, are also suitable for parkinsonian rodent models induced by other neurotoxins such as rotenone [6], lipopolysaccharide (LPS) [7], and 1-methyl-4-phenyl-1,2,3,6-tetrahydropyridine (MPTP) [8]. Also for other pathological entities, in which movement disorders occur, such as the following: amyotrophic lateral sclerosis [9], Huntington's disease [10], cerebral stroke [11,12], intracerebral hemorrhage [13], and spinal injury [12]. The methodology of behavioral tests presented here underwent some modifications to the original protocol.

**Step 1: Unilateral intranigral or striatal injury by stereotaxic administration of a neurotoxin in the rat.**

### Materials

• Adult male *Wistar* rats (210–230 g)
• Ketamine
• Xylazine
• Stereotaxic apparatus
• Dimethylsulfoxide (DMSO)
• BSSG
• 6-OHDA
• Phosphate-buffered saline solution (PBS)
• Ascorbic acid
• Microperfusion pump
• Silk 00
• Oxytetracycline and polymyxin B
• Evans blue dye
• 95% of ethanol

### Ethical statements

The experiments were approved by the Institutional Committee for the Care and Use of Laboratory Animals of the Center for Research and Advanced Studies of the National Polytechnic Institute (CINVESTAV-IPN) and regulated under the Official Mexican Standard NOM-062-ZOO-1999. Adult male *Wistar* rats (210–230 g) were obtained from the Laboratory of Animal Production and Experimentation Unit of CINVESTAV-IPN (Protocol 162–15).

### Procedure

1. Animals were anesthetized with a mixture of 100 mg/kg ketamine and 10 mg/kg xylazine injected intraperitoneally.
2. They were placed on a stereotaxic frame (Stoelting; Wood Dale, IL, USA), and the skull was trepanned with the following coordinates for BSSG administration in the left substantia nigra pars compacta (SNpc): anteroposterior (AP), +2.1 mm from interaural midpoint; medial-lateral (ML), +2.0 mm from interparietal suture; and dorsal-ventral (DV), −6.8 mm from dura mater [3,14]. The coordinates for 6-OHDA administration in the left striatum were: AP, +7.7 mm; ML, +4.0 mm; and DV, −5.4 [15].

**Note:** It is important that the dura mater is broken for the correct neurotoxin diffusion. It can be done with the tip of a hypodermic needle.

3. BSSG (Daucosterol; MedChemExpress) was administered at a concentration of 6 µg/µl DMSO (Sigma- Aldrich; St. Louis, MO, USA), or only 1 µl of DMSO (mock group). The concentration of 6-OHDA (Sigma- Aldrich; St. Louis, MO, USA) was 20 µg free base in 3 µL of PBS containing 0.2% ascorbic acid (Sigma-Aldrich; St. Louis, MO, USA). These neurotoxins were independently injected using a microperfusion pump (Stoelting; Wood Dale, IL, USA) to maintain a flow rate of 0.13 µL/min.

**Note:** We recommend using a blunt 20-gauge dental needle, so that the diffusion of the neurotoxin remains in a radial direction, conversely to the bevel orientation. Once the infusion is finished, wait between 5 and 10 min to allow the complete and appropriate neurotoxin diffusion.

4. The wound was sutured with silk 00 and treated with a mixture of oxytetracycline and polymyxin B (Pfizer; Toluca, Mexico) to prevent infections.

**Note:** Before performing stereotaxic surgeries, it is a good practice to verify the coordinates and the lesion site by dye-staining, i.e., by injecting Evans blue dye (Sigma-Aldrich; St. Louis, MO, USA) in 95% of ethanol.

**Step 2: Behavioral tests of parkinsonian rodents**

Depending on the neurotoxin, the behavioral tests begin when the dopaminergic lesion of the nigrostriatal system elicits measurable motor behavior impairments. For example; the effect of BSSG can be evaluated from day 15 after the lesion [3] and that of 6-OHDA from 2 to 3 weeks post-lesion [4].

Rodents can be housed individually or in groups; however, once defined the scheme, it should be maintained to avoid behavior changes. Since rodents are social animals, single housing may affect the results in several behavioral tests validated herein. Undesirable effects from isolation are anxiety-like behavior, changes in neurochemical functions, and hyperactivity [16]. On this basis, it is preferable to use group housing. Also, rodents should be daily handled for a few minutes for habituation purposes (at least 5 days before the behavioral tests), since stress can modify the results.

In all behavioral tests, between each evaluation, the devices and surfaces must be cleaned with 30% ethanol. In the forced swimming test, the water should be changed after each evaluation to discard the influence of odors, substances and temperature.

The six behavioral tests can be performed over the course of a week. Moreover, to prevent animals from stressing and behavioral interference between one test and another, it is recommended the following sequence:

Day 1: Open field test.
Day 2: Vibrissae test. Olfactory asymmetry pre-test.
Day 3: Olfactory asymmetry test.
Day 4: Uncoordinated gait test.
Day 5: Cylinder test.
Day 6: Forced swim pre-test.
Day 7: Forced swim test.

On days 2 and 3, a training will be required for the uncoordinated gait test, which will be evaluated on day 4. This training should be conducted 2 h before the evaluation of the indicated tests on those days. On day 6 and 7, do not perform any other behavioral tests, since water stresses animals.

**Note:** If it is intended to conduct a behavioral assessment in a temporal course, tests must be performed with at least 45 days inter-trial to avoid habituation to the next test, since animals generally become accustomed to their environment. If it is not possible to wait for that period, it is recommendable to use a new group of animals for each time point.

**Step 2.1: Open field test (day 1)**

### Materials

**Fig. 1:**
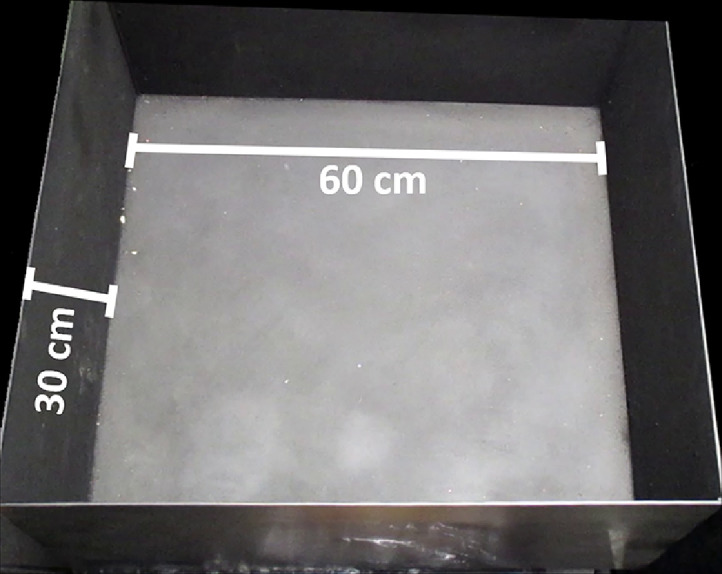
Properties of the open field box. It is a square wooden box, with the following dimensions: 60 cm wide, 60 cm long and 30 cm high.
• Method for acquiring the data: It can be done manually or by computerized tracking software. Our evaluation was done through the Videomex-V automated system.
• Alternative video camera.
• Timer: This may be tracked by the apparatus/software or by a separate handheld timer.
• Material for clean-up: It usually includes a dumpster and paper towels to clean urine and feces.
• Disinfectant: We recommend using alcohol between 10% and 50% for general cleaning.

• Open field: The material of the box can be plastic, metal, or wood, that could be easily cleaned and sterilized. The recommended characteristics for the box include a square box of 60 cm width and 30 cm high per wall (Fig. 1) for both, rats and mice.

### Procedure

1. Move the rodents to the test room at least 1 h before the test, to allow animals to acclimatize to the experimental room.

**Note:** A room isolated from sound and involuntary interruptions should be used. If there is vulnerability to this, white noise can be used. Also, we advise using a dim light in the room, since a bright white light tends to decrease locomotion.

2. Turn on the apparatus or software.

**Note:** It is advisable to record each session to allow re-evaluation of results, if necessary.

3. Before placing the animal into the box, clean it to remove any residual odor.
4. Place the rodent next to a wall for the intended time.

**Note:** Placing the rodent in the center of the box can subject it to an immediate stress situation.
The evaluation time can vary; generally, it is 5 min to 1 h. We evaluated the locomotor activity during 9 min and observed that the first 3 min embrace the exploratory activity and the next 6 min allow the identification of locomotor alterations (bradykinesia, Supplementary video 1) in the rodent.

5. At the end of the test, remove the animal from the open field and place it in a new cage. Do not join evaluated rodents with those to be assessed, to avoid inducing them stress.
6. Clean the box with the disinfectant and wait until it has completely dried, before placing a new rodent.
  **Note:** In addition to the locomotor activity, the emotional or anxiety state of the animal can be analyzed through the number of fecal pellets made at the end of the test.

The spontaneous locomotor activity (Supplementary video 1) can be analyzed by the open field test when animals are exposed to a new environment [17,18]. The distance traveled (in cm) was measured by an automated system (Videomex-V) for 9 min. This software provides the distance traveled by the rodent, as well as the resting and walking times. Because these three variables are directly proportional to each other and yield the same information, any of them can be used.

**Step 2.2: Vibrissae test (day 2)**

### Materials

• Edge of a table.
• Two video cameras mounted on a tripod.
• Material for clean-up: A dumpster and paper towels to clean urine and fecal pellets.
• Disinfectant: Alcohol between 10% and 50% for surfaces cleaning.
• A new cage.

### Procedure

1. Transfer the animals to the room where the behavioral test will be conducted at least 1 h before the test, to allow the animals to acclimatize.

**Note:** A room isolated from sound and involuntary interruptions is recommended, and if necessary, white noise.

**Fig. 2:**
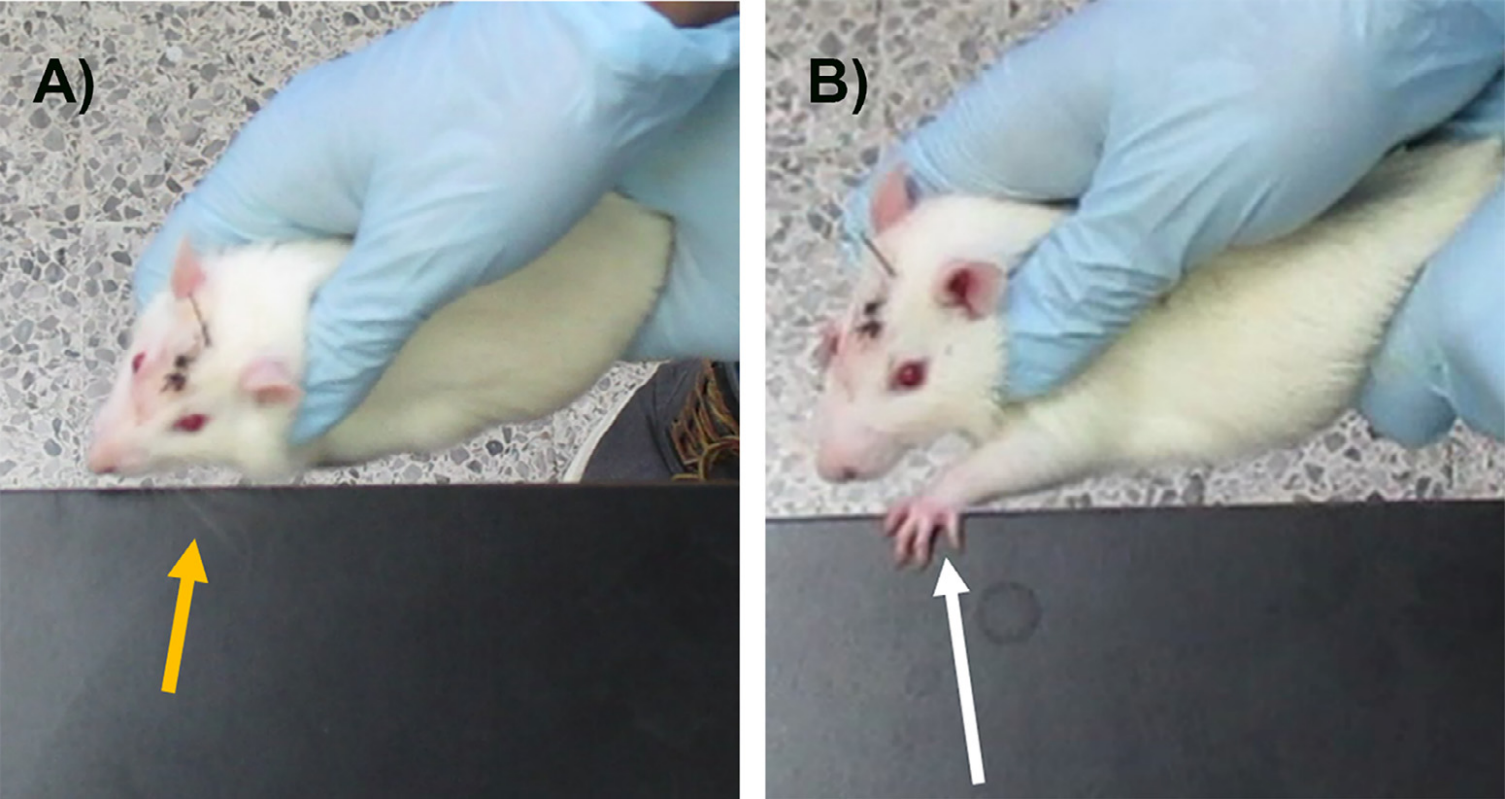
Vibrissae test. Panel A) shows the stimulation of the vibrissae against the edge of table (orange arrow). Panel B) shows the ipsilateral forelimb placement reflex (white arrow).

**Note:** It is essential to firmly hold the rodent torso, avoiding the obstruction of the fore or hind limb of the rodents by the researcher´s hand.

2. Clean up the surface with disinfectant before and after each behavioral test.
3. Turn on the video cameras and place them alongside and directly above the edge of the table to ensure a sharp and clear scene. The video recording will allow further evaluation and distinction of individual behaviors (Supplementary video 2).
4. Animals must be gently held through the torso (Fig. 2).

5. The vibrissae are stimulated by brushing against the edge of a table (Fig. 2a), to elicit an ipsilateral forelimb response that consists in placing the paw on the tabletop (Fig. 2b, Supplementary video 2).

**Note:** Consider that trials in which the animal struggles, must not be counted.
Take into consideration that this test is suitable for ipsilateral evaluation. However, if a bilateral affection exists, both sides should be evaluated.

6. After the test, it is usually preferable to place the animal in a new cage, rather than put it back with its cage mates, as the reintroduction of the rodent may modify the behavior of the untested animals.

The response of healthy animals consists of a quick placement of their forelimb on the table surface after vibrissae stimulation. The absence or a decrease in the response displays a deficit of motor behavior (Supplementary video 2), rather than a sensory impairment [4,19]. The number of successful forelimb placements contralateral and ipsilateral to the lesion is counted in a maximum of 10 events.

**Step 2.3: Olfactory asymmetry (days 2 and 3)**

### Materials

**Fig. 3:**
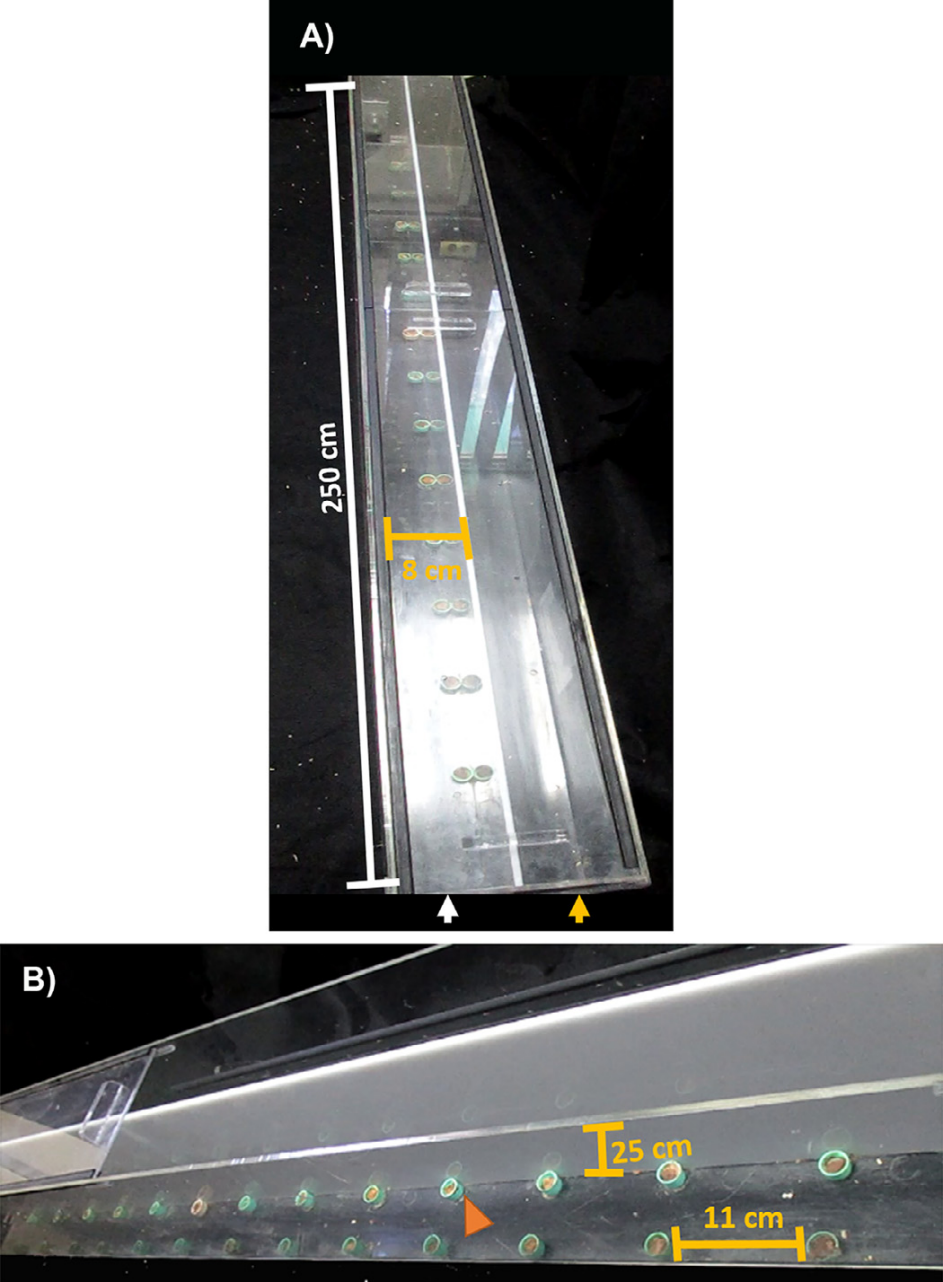
Corridor properties. A) Top view of a 250-cm long acrylic corridor showing the training compartment (orange arrow) and the test compartment (white arrow) of 8-cm wide each one. B) Lateral-superior view showing the 25-cm high acrylic wall that separates each compartment. On the corridor floor, the plastic caps containing the chocolate pellets (orange arrowhead) can be observed with a separation of 11 cm from each other.
• Plastic caps (2 cm diameter and 1 cm deep): they were placed along the floor of the testing compartment (fixed with glue), symmetrically aligned at left and right sides. Caps were separated by 11 cm along the corridor (Fig. 3b) for rat evaluation, and 5 cm for mice testing.
• Chocolate pellets: They are firmly placed within the plastic caps in both compartments of the corridor (Fig. 3b).

**Note:** Sugar pellets are recommended by other protocols [20]; however, chocolate pellets are more attractive for rodents.

• The corridor is a long rectangular box (250 cm long, 8 cm wide and 25 cm high for rat evaluation) of acrylic material. Since this test has not been used in mice, we propose a box of 150 cm long, 4 cm wide and 15 cm high for evaluating them. Any box has a sliding roof with perforations for airflow (Fig. 3a) and two parallel compartments (separated by a white acrylic wall; Fig. 3b); one of them for habituating animals, and another for behavioral testing (Fig. 3a).

• Two video cameras mounted on their respective tripod.
• Timer: registered by a handheld timer or video cameras.
• Material for clean-up: Paper towels to clean fecal pellets and urine.
• Disinfectant: Alcohol between 10% and 50% for cleaning corridor surfaces.
• A new cage.

### Procedure

1. Transfer animals to the behavioral room, at least 1 h before the test, to secure their acclimatization.
2. One day before the test, the animals should be exposed to chocolate pellets in the habituation compartment for 10 min to reduce the exploratory behavior.

**Note:** Place chocolate pellets at the habituation compartment ends.

3. Throughout habituation and testing, maintain at 90% of free-feeding body weight.
4. On the next day, repeat the step 1, place again the rodent in the habituation compartment and count 10 min by a handheld timer.
5. Before placing the rodent in the testing compartment, turn on the video cameras.

**Note:** Place one of the video cameras above the corridor and the other one in the frontal end.

6. Subsequently, place the animal in the testing compartment for 5 min.

**Note:** In addition to video recording, we recommend registering details of rodent's behavior such as numbers of touches with the nose on the chocolate pellet, in a logbook.

7. After the behavioral test, place the rodent in a new cage to avoid stressing their non-evaluated cage mates.
8. Be sure to disinfect the corridor surfaces between each animal evaluation.

The test concludes after a total of 20 touches with the tip of their nose over the pellet (Supplementary video 3) or after a maximum test time of 5 min [20,21]. The percentage of olfactory asymmetry is evaluated as follows:

$$\%\;\text{of}\;\text{asymmetry}\;=\frac{\text{number}\;\text{of}\;\text{contralateral}\;\text{touches}\;\text{to}\;\text{the}\;\text{lesion}}{\text{number}\;\text{of}\;\text{touches}\;\text{contralateral}\;+\;\text{ipsilateral}\;\text{to}\;\text{the}\;\text{lesion}}x\;100$$

**Step 2.4: Uncoordinated gait test (day 4)**

### Materials

**Fig. 4:**
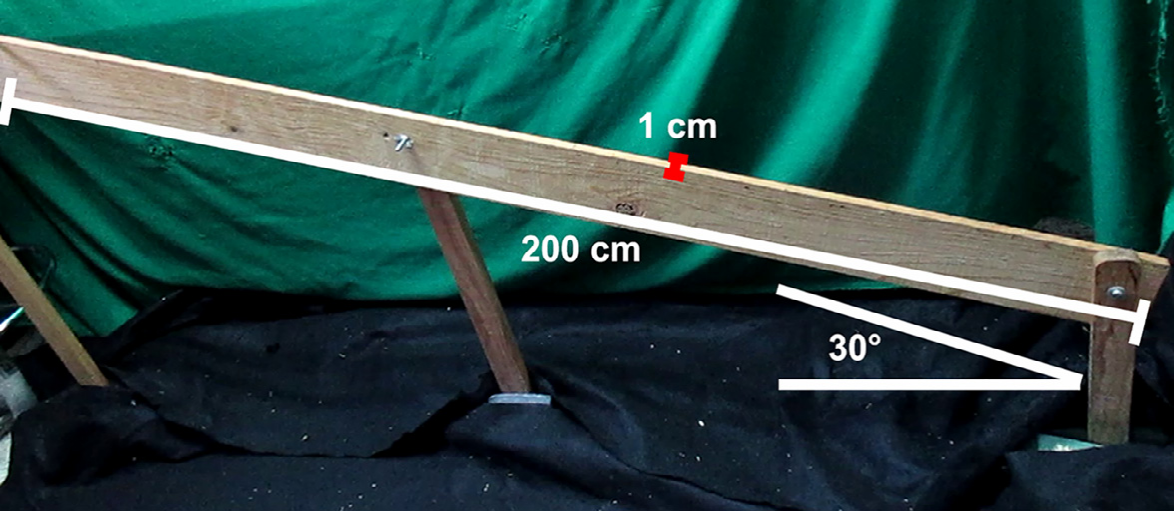
Beam features. The beam is made of wood and is firmly attached to the floor with the following dimensions, 200 cm length, 1 cm wide and a 30° angle.

**Note:** The beams must be at a 30-degree angle to challenge the rat balance and coordination (Fig. 4).

• Two narrow wooden beams for rat evaluations: (1) 1 cm wide and 2 m long, and (2) 2 cm wide and 2 m long (Fig. 4).

• Two narrow wooden beams for mouse evaluations: (1) 30 mm wide and 1 m long, and (2) 10 mm wide and 1 m long.
• **Note:** The beams must be placed horizontally at 50 cm from the floor.
• Two video cameras mounted on their respective tripod.
• Timer: This may be tracked by the video cameras or by a separate handheld timer.
• Material for clean-up: Paper towels to clean urine and fecal pellets.
• Disinfectant: Alcohol between 10% and 50% for surfaces cleaning.
• A new cage.

### Procedure

We standardized this behavioral test for rat evaluation for the first time, since all protocols have been developed for the mouse model [10,22].

1. Move the animals to the behavioral room, at least 1 h before the test for acclimatization.

**Note:** A room isolated from sound is recommendable, even white noise, if necessary.

2. Clean up the beam surface with disinfectant before and after each test.
  **Note:** The beam must be held firmly attached to the floor to avoid influencing the animal´s balance.
3. On the first day of the pre-test, place a new cage with sawdust at the upper end of the beam; then, place the rodent at its lower end (Supplementary video 4).
  **Note:** Only on this day use the thickest beam (2 cm wide for rats and 30 mm wide for mice).
4. Wait until the rodent walks to the upper end of the beam.
  **Note:** Generally, on this first day, it is complicated for animals to walk through the beam. Impulse the rodent by gently pushing from its hind limbs. For this behavioral test, two people should help to perform it.
5. Train the animals again 24 h later, but now using the thinner beam (1 cm wide for rat and 10 mm wide for mice) and repeat steps 1–4.
6. On the next day, the final test will be performed on the thinner beam (1 cm wide for rat and 10 mm wide for mice). Be sure to turn on the video cameras and place them beside and in front of the beam.
  **Note:** A lateral view of the beam adequately allows demonstrating the rodent slips (Fig. 4, Supplementary video 4). The precise point/moment to measure the slips or errors per step is when both hind limbs are not properly supported on the beam and slide off (1 cm for rats and 0.5 cm for mice) toward the lateral side of the beam.

In addition to the time recorded by the video camera, the walking time should be registered with a handheld timer.

The beam walking test measures the ability of rodents to displace on a narrow beam. This test evaluates the balance and motor coordination during the displacement time (Supplementary video 4), and the number of slips of the hind limbs (Supplementary video 4). Hind limbs undoubtedly provide the most sensitive measure of performance and motor deficits [10].

**Step 2.5: Cylinder test (day 5)**

### Materials

**Fig. 5:**
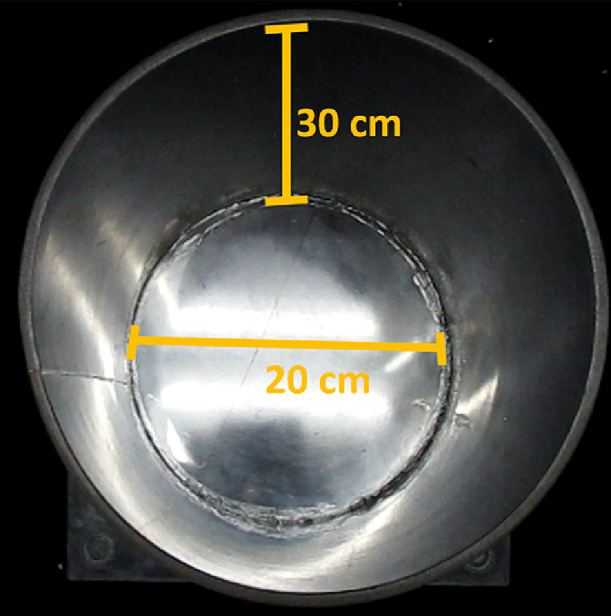
Cylinder characteristics to assess locomotor asymmetry in the rat. It is made of acrylic material and has the following dimensions: 30 cm height and 20 cm in diameter.
• An acrylic cylinder of 15 cm high and 10 cm diameter for mouse evaluations.
• A video camera mounted on its tripod.
• Material for clean-up: Paper towels to clean urine and fecal pellets.
• Disinfectant: Alcohol between 10% and 50% for surfaces cleaning.
• A new cage.

• An acrylic cylinder of 30 cm high and 20 cm diameter for rat evaluations (Fig. 5).

### Procedure

1. Animals must remain in the behavioral room at least 1 h before the test.
2. Disinfect cylinder surfaces before and after each behavioral test.

**Note:** Like the open field test does, the number of fecal pellets evidences the emotional state and level of stress of animals.

3. Place and turn on the video camera above the cylinder.
4. Place the rodent inside the cylinder and remove it after it makes 20 contacts with its forelimbs on the cylinder walls (Supplementary video 5).

**Note:** Rodents generally make 20 contacts within the first 3 min. However, the time may be longer due to akinesia or bradykinesia, which can be more accurately evaluated by other tests, such as the stepping [23], the open field and the pole tests [24].

The cylinder test is appropriate to evaluate locomotor asymmetry [4,^15^,18]. The experimenter should count the first 20 contacts made on the cylinder wall with both, the ipsilateral or contralateral paws to the lesion (Supplementary video 5). The time required to generate this number of observations varies from animal to animal. The percentage of locomotor asymmetry is calculated as follows [4]:

$$\%\;\text{of}\;\text{asymmetry}\;=\frac{\text{contacts}\;\text{of}\;\text{ipsilateral}\;\text{forelimb}+\frac{1}{2}\;\text{simultaneous}\;\text{contacts}}{\text{ipsilateral}+\text{contralateral}+\text{simultaneous}\;\text{contacts}}x\;100$$

The ipsilateral and contralateral contacts are related to the lesion side. The simultaneous contacts were counted when both forelimbs were on the cylinder wall at the same time, and are halved only to consider the contacts with the ipsilateral forelimb. The divider corresponds to the total number of contacts observed. A value of 50% indicates that an animal symmetrically explores with both forelimbs. Higher scores (> 50%) indicate less dependence on contralateral forelimb (since it evaluates the injured side), and lower scores (< 50%) indicate less dependence on the ipsilateral forelimb (since it evaluates the control side).

**Step 2.6: Forced swim test (days 6 and 7)**

### Materials

**Fig. 6:**
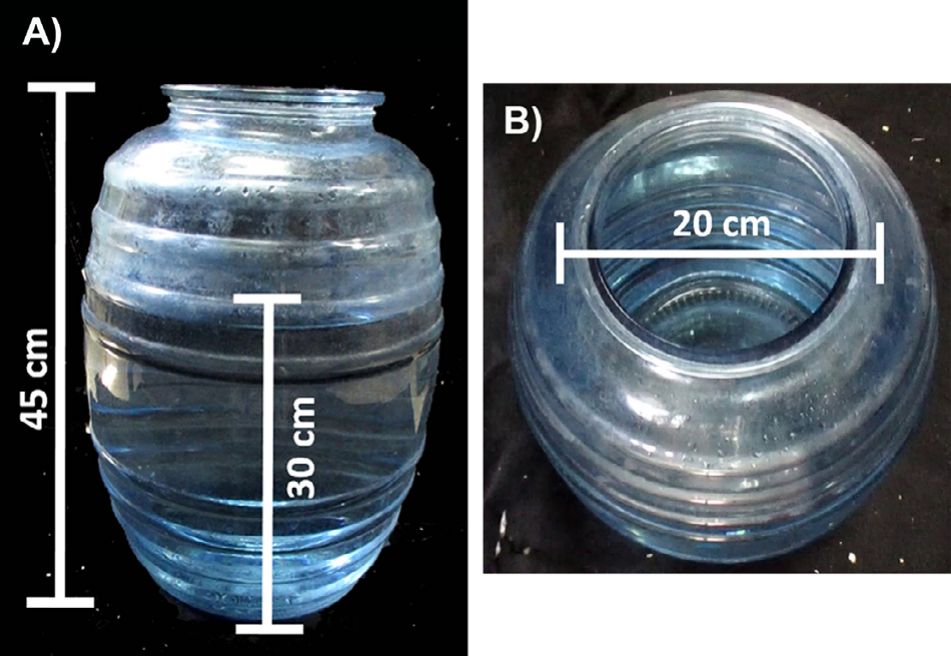
Swim cylinder characteristics. A) The lateral view shows the high of the cylinder (45 cm) and the water depth (30 cm). B) The top view shows the cylinder diameter (20 cm).
• Mercury thermometer (to check water temperature).
• Two video cameras mounted in their respective tripod.
• Microfiber towels (to dry animals).
• A lamp.

• Swim cylinders (usually between 1 and 4) of 20 cm diameter and 45 cm high for rats (Fig. 6) or 20 cm diameter and 30 cm high for mice.

### Procedure

1. On the pre-test day, animals must be transferred to the room where the test will be carried out at least 1 h before, to avoid stress.
2. Fill in the swimming cylinders with water at a depth of approximately 30 cm (Fig. 6a) for rats and 15 cm for mice.
3. The temperature must be between 22–24 °C. Water temperature can be monitored with a mercury thermometer.
  Note: The range of temperature between 22 and 24 °C is habitual in most laboratories and allows to maintain a constant temperature for water. Lower temperatures could lead to animals’ hypothermia, possibly affecting their activity [25].
4. Place animals individually in the swim cylinders for 15 min. After this time, remove rodents from water and dry them as best as possible with microfiber towels. Return them to their cages, providing heat through a lamp.
  **Note:** If rodents are housed in a group, those subjected to the swim pre-test should not be mixed with unevaluated rodents, since their encounter can cause them stress. Therefore, it is advisable to house the animal temporary (2 h) in a separate cage, and then regroup it according to the experimental condition.
5. Water changes between each evaluation must be done, keeping the same depth and temperature, as mentioned above.
6. Refill the swim cylinders 24 h after the pre-test, before placing rodents in them. Start the video recording, ensuring the full duration of the test.
  **Note:** We recommend recording the test directly, above and alongside the swim cylinders. Also, ensure image quality (clarity and sharpness) to allow proper evaluation and distinction of individual behaviors.
7. Place the rodents in the swim cylinder for 5 min. After this time has passed, dry animals and put them back in their cages.
  **Note:** A lateral view of the swim cylinder adequately evidences rodent immobility behavior (Supplementary video 6).

A depressive-like behavior can be measured in rodents when they are exposed to water in a swim cylinder [25]. In this paradigm, the first reaction is trying to escape by climbing or swimming, but later, they will acquire an immobility behavior (Supplementary video 6). This behavior reflects a failed attempt to escape and deal against the stress [25,26].

## Appendix Supplementary materials

Image, video 1

Image, video 2

Image, video 3

Image, video 4

Image, video 5

Image, video 6

## References

[1] Shen W.B., McDowell K.A., Siebert A.A., Clark S.M., Dugger N.V., Valentino K.M., Jinnah H.A., Sztalryd C., Fishman P.S., Shaw C.A., Jafri M.S., Yarowsky P.J. (2010). Environmental neurotoxin-induced progressive model of parkinsonism in rats. Ann. Neurol..

[2] Van Kampen J.M., Baranowski D.C., Robertson H.A., Shaw C.A., Kay D.G. (2015). The progressive bssg rat model of Parkinson’s: recapitulating multiple key features of the human disease. PLoS One.

[3] Soto-Rojas L.O., Garces-Ramirez L., Luna-Herrera C., Flores-Martinez Y.M., Soto-Rodriguez G., Gatica-Garcia B., Lopez-Salas F.E., Ayala-Davila J., Gutierrez-Castillo M.E., Padilla-Viveros A., Banuelos C., de la Cruz-Lopez F., Martinez-Davila I.A., Martinez-Fong D. (2020). A single intranigral administration of beta-sitosterol beta-d-glucoside elicits bilateral sensorimotor and non-motor alterations in the rat. Behav. Brain Res..

[4] Woodlee M.T., Kane J.R., Chang J., Cormack L.K., Schallert T. (2008). Enhanced function in the good forelimb of hemi-parkinson rats: compensatory adaptation for contralateral postural instability?. Exp. Neurol..

[5] Simola N., Morelli M., Carta A.R. (2007). The 6-hydroxydopamine model of Parkinson’s disease. Neurotox. Res..

[6] Moreira C.G., Barbiero J.K., Ariza D., Dombrowski P.A., Sabioni P., Bortolanza M., Da Cunha C., Vital M.A., Lima M.M. (2012). Behavioral, neurochemical and histological alterations promoted by bilateral intranigral rotenone administration: a new approach for an old neurotoxin. Neurotox. Res..

[7] Flores-Martinez Y.M., Fernandez-Parrilla M.A., Ayala-Davila J., Reyes-Corona D., Blanco-Alvarez V.M., Soto-Rojas L.O., Luna-Herrera C., Gonzalez-Barrios J.A., Leon-Chavez B.A., Gutierrez-Castillo M.E., Martinez-Davila I.A., Martinez-Fong D. (2018). Acute neuroinflammatory response in the substantia nigra pars compacta of rats after a local injection of lipopolysaccharide. J. Immunol. Res..

[8] Santiago R.M., Barbieiro J., Lima M.M., Dombrowski P.A., Andreatini R., Vital M.A. (2010). Depressive-like behaviors alterations induced by intranigral MPTP, 6-OHDA, lps and rotenone models of Parkinson's disease are predominantly associated with serotonin and dopamine. Prog. Neuropsychopharmacol. Biol. Psychiatry.

[9] Tabata R.C., Wilson J.M., Ly P., Zwiegers P., Kwok D., Van Kampen J.M., Cashman N., Shaw C.A. (2008). Chronic exposure to dietary sterol glucosides is neurotoxic to motor neurons and induces an als-pdc phenotype. Neuromol. Med..

[10] Carter R.J., Lione L.A., Humby T., Mangiarini L., Mahal A., Bates G.P., Dunnett S.B., Morton A.J. (1999). Characterization of progressive motor deficits in mice transgenic for the human Huntington's disease mutation. J. Neurosci..

[11] Schaar K.L., Brenneman M.M., Savitz S.I. (2010). Functional assessments in the rodent stroke model. Exp. Transl. Stroke Med..

[12] Schallert T., Fleming S.M., Leasure J.L., Tillerson J.L., Bland S.T. (2000). CNS plasticity and assessment of forelimb sensorimotor outcome in unilateral rat models of stroke, cortical ablation, parkinsonism and spinal cord injury. Neuropharmacology.

[13] Hua Y., Schallert T., Keep R.F., Wu J., Hoff J.T., Xi G. (2002). Behavioral tests after intracerebral hemorrhage in the rat. Stroke.

[14] Hernandez-Baltazar D., Mendoza-Garrido M.E., Martinez-Fong D. (2013). Activation of GSK-3beta and caspase-3 occurs in nigral dopamine neurons during the development of apoptosis activated by a striatal injection of 6-hydroxydopamine. PLoS One.

[15] Reyes-Corona D., Vazquez-Hernandez N., Escobedo L., Orozco-Barrios C.E., Ayala-Davila J., Moreno M.G., Amaro-Lara M.E., Flores-Martinez Y.M., Espadas-Alvarez A.J., Fernandez-Parrilla M.A., Gonzalez-Barrios J.A., Gutierrez-Castillo M.E., Gonzalez-Burgos I., Martinez-Fong D. (2017). Neurturin overexpression in dopaminergic neurons induces presynaptic and postsynaptic structural changes in rats with chronic 6-hydroxydopamine lesion. PLoS One.

[16] Voikar V., Polus A., Vasar E., Rauvala H. (2005). Long-term individual housing in C57BL/6J and DBA/2 mice: assessment of behavioral consequences. Genes Brain Behav..

[17] Hernandez-Chan N.G., Bannon M.J., Orozco-Barrios C.E., Escobedo L., Zamudio S., De la Cruz F., Gongora-Alfaro J.L., Armendariz-Borunda J., Reyes-Corona D., Espadas-Alvarez A.J., Flores-Martinez Y.M., Ayala-Davila J., Hernandez-Gutierrez M.E., Pavon L., Garcia-Villegas R., Nadella R., Martinez-Fong D. (2015). Neurotensin-polyplex-mediated brain-derived neurotrophic factor gene delivery into nigral dopamine neurons prevents nigrostriatal degeneration in a rat model of early Parkinson's disease. J. Biomed. Sci..

[18] Balkaya M., Krober J.M., Rex A., Endres M. (2013). Assessing post-stroke behavior in mouse models of focal ischemia. J. Cereb. Blood Flow Metab..

[19] Barth T.M., Stanfield B.B. (1994). Homotopic, but not heterotopic, fetal cortical transplants can result in functional sparing following neonatal damage to the frontal cortex in rats. Cereb. Cortex.

[20] Dowd E., Monville C., Torres E.M., Dunnett S.B. (2005). The corridor task: a simple test of lateralised response selection sensitive to unilateral dopamine deafferentation and graft-derived dopamine replacement in the striatum. Brain Res. Bull..

[21] Boix J., Padel T., Paul G. (2015). A partial lesion model of Parkinson's disease in mice–characterization of a 6-OHDA-induced medial forebrain bundle lesion. Behav. Brain Res..

[22] Luong T.N., Carlisle H.J., Southwell A., Patterson P.H. (2011). Assessment of motor balance and coordination in mice using the balance beam. J. Vis. Exp..

[23] Cordeiro K.K., Cordeiro J.G., Furlanetti L.L., Garcia Salazar J.A., Tenorio S.B., Winkler C., Dobrossy M.D., Nikkhah G. (2014). Subthalamic nucleus lesion improves cell survival and functional recovery following dopaminergic cell transplantation in parkinsonian rats. Eur. J. Neurosci..

[24] Sedelis M., Schwarting R.K., Huston J.P. (2001). Behavioral phenotyping of the mptp mouse model of Parkinson’s disease. Behav. Brain Res..

[25] Slattery D.A., Cryan J.F. (2012). Using the rat forced swim test to assess antidepressant-like activity in rodents. Nat. Protoc..

[26] Slattery D.A., Desrayaud S., Cryan J.F. (2005). GABAB receptor antagonist-mediated antidepressant-like behavior is serotonin-dependent. J. Pharmacol. Exp. Ther..

